# Numerical Study on the Heat Transfer of Carbon Dioxide in Horizontal Straight Tubes under Supercritical Pressure

**DOI:** 10.1371/journal.pone.0159602

**Published:** 2016-07-26

**Authors:** Mei Yang

**Affiliations:** Key Laboratory of Low-grade Energy Utilization Technologies and Systems, Ministry of Education, College of Power Engineering, Chongqing University, Chongqing, China; Tsinghua University, CHINA

## Abstract

Cooling heat transfer of supercritical CO_2_ in horizontal straight tubes with wall is numerically investigated by using FLUENT. The results show that almost all models are able to present the trend of heat transfer qualitatively, and the stand *k*−*ε* with enhanced wall treatment model shows the best agreement with the experimental data, followed by LB low Re turbulence model. Then further studies are discussed on velocity, temperature and turbulence distributions. The parameters which are defined as the criterion of buoyancy effect on convection heat transfer are introduced to judge the condition of the fluid. The relationships among the inlet temperature, outlet temperature, the mass flow rate, the heat flux and the diameter are discussed and the difference between the cooling and heating of CO_2_ are compared.

## 1. Introduction

CO_2_ has zero ozone depletion potential (ODP) and zero global warming potential (GWP).The critical temperature CO_2_ is relatively low and about 31.1°C. It is close to the ambient temperature. The systems with CO_2_ operating at ambient temperature are likely to work close to the critical pressure of 7.38 MPa.

Recently there are many studies about the heat transfer of heating and cooling of supercritical CO_2_ in the tube. Liao and Zhao[1] experimentally investigated the convective heat transfer of supercritical CO_2_ in horizontal and vertical tube. The diameter of the tube was 0.7mm, 1.4mm and 2.16mm, and they found that the buoyancy still had an important influence on convective heat transfer of supercritical CO_2_ although Re had reached 10^5^,.

Jiang et al.[2] [3]studied the convective heat transfer of supercritical CO_2_ using experimental and numerical method in vertical micro channel with diameter 0.27mm and they found that the effect of flow direction, buoyancy and self-acceleration on the wall temperature were not big. The heat transfer coefficient initially increased with increasing heat flux and then decreased with further increase in the heat flux for both upward and downward flows. When the heat flow was relatively large, the buoyancy effect was very low. However, the acceleration caused by the buoyancy affected the turbulent kinetic energy and made the heat transfer coefficient become smaller in the large heat flow rate.

Dang et al.[4] experimentally investigated the heat transfer coefficient and pressure drop of four horizontal cooling tubes with different inner diameters ranging from 1 to 6 mm. The effects of mass flux, pressure, and heat flux were measured and the correlation was proposed according to the experimental data.

Du [5] simulated the cooling heat transfer of the supercritical CO_2_ by numerical method in horizontal straight tube with diameter 6mm, and found that almost all of the turbulence models could simulate cooling heat transfer of the supercritical CO_2_ in a straight tube. Yang et al.[6] studied the cooling heat transfer of supercritical CO_2_ using numerical method in the straight tube. The parameters of the tube were: diameter 0.5mm, length 1000mm, seven different tilt angles and they found that the heat transfer of the horizontal straight tube had the best effect.

Liu et al.[7] experimentally investigated the heat transfer and pressure drop performances of CO_2_ cooled in horizontal tubes having inner diameters of 4, 6 and 10.7 mm. The results showed that the tube diameter significantly affected the heat transfer performance. Based on the experimental data, a new heat transfer correlation was proposed for the large diameter tube.

Lei et al.[8] numerically investigated the mechanism of heat transfer phenomena of water in horizontal smooth tubes under supercritical pressures. Both the heat transfer enhancement and heat transfer deterioration in the large specific heat region of supercritical fluids were analyzed. It was showed that in the large specific heat region, there existed strong non uniformity in the circumferential distribution of the tube inner wall temperature which was mainly due to the rapid change in fluid properties.

Yu et al.[9] experimentally studied the effect of buoyancy on heat transfer characteristics of supercritical pressure water in horizontal tubes with inner diameters of 26 mm and 43 mm. It was found that the buoyancy effect made the low density hot water gather at the top surface of the horizontal tube, hence heat transfer condition was deteriorated and wall temperature increased.

In this paper the heat transfer of the supercritical CO_2_ in horizontal straight tubes is numerically simulated and the most suitable numerical simulation methods are investigated according to comparing the simulation values with the experimental values. The effects of different heat flux, mass flow rate, tube length, tube diameter and inlet temperature on heat transfer coefficient are analyzed and the relevant conclusions are obtained. The dimensionless numbers which are used to define the effect of the buoyancy are calculated.

## 2. Material and Methods

### 2.1. Numerical method and boundary condition

The simulation condition is cooling heat exchanger of supercritical CO_2_ in horizontal straight tube with constant heat flux. The pressure is equal to 8.0 MPa, and the *C*_*p*_ reaches a peak value at the pseudo-critical temperature (*T*_*pc*_) of 307.6 K. The properties of supercritical CO_2_ are used to calculate in FLUENT software by defining a piecewise-linear function of temperature and the NIST Standard Reference Database 23 is used to determine the thermodynamic properties.

The models that predict accurately the heat transfer under heating conditions will be used for studying cooling heat transfer of supercritical CO_2_, including standard *k*−*ε*, RNG *k*−*ε*, *k*−*ω* model and six low Re turbulence models: AB[10], LB[11], LS[12], YS[13], AKN[14] and CHC[15]. All these models are operated

The FLUENT 6.3 is used in the numerical calculation. The boundary condition is set as: the mass flow inlet boundary, the outflow boundary and the constant heat flux wall. SIMPLE algorithm is used in pressure-velocity coupling equation. The specific dissipation rate equation and turbulent kinetic energy equation use the first order upwind, and momentum equation and energy equation adopt the second order upwind. When the relative residual for each governing equation is less than 10^−5^, the numerical calculation is considered converged.

### 2.2. Physical model and meshing display

**Fig 1:**
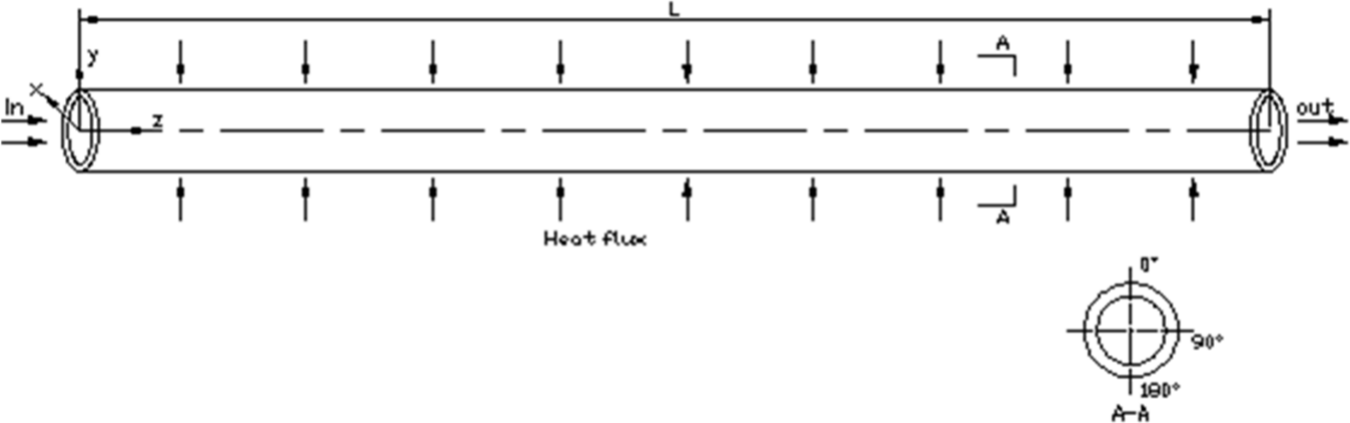
Physical model of horizontal straight tube.

Physical model of the horizontal tube is as shown below in Fig 1. The inner diameter of the tube is 6/10.7/27 mm with a wall thickness of 1/2/3 mm and the length is 500/1300/8000 mm. The circumferential angle at the top is defined as 0°and the bottom is 180°.

**Fig 2:**
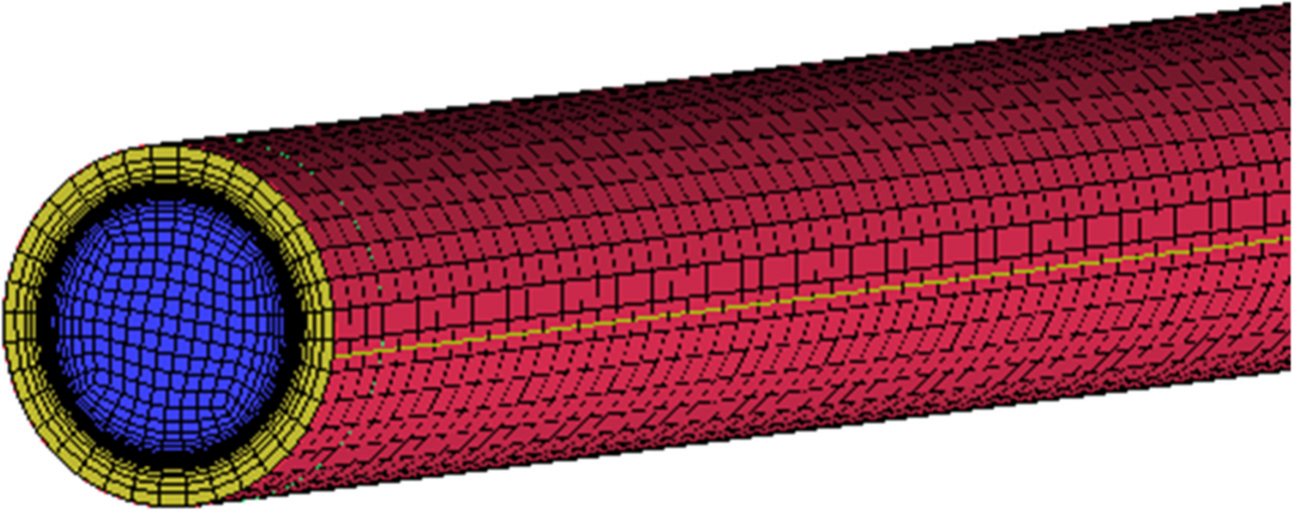
The grid of horizontal straight tube.

Fig 2 shows the grid schematic view of horizontal straight tube. The grid is generated by the ICEM software. The mesh quality requires more than 0.2 and the quality of grid using in this paper is larger than 0.5 to meet the requirement. The dimensionless distance y+ requires less than one and the present grid meets the requirement.

## 3. Results and Discussion

### 3.1. Model selection

Selection of turbulence model plays an important role in numerical simulation. The simulated conditions performed in this passage are that mass flux is 200 kg/m^2^, the inlet pressure at 8MPa, the inlet temperature at 330 K and the heat flux at 33 kW/m^2^.These conditions are the same with the experimental conditions of Dang and Hihara[4]. The same condition was calculated by Du et al[5]. Using some turbulence models to simulate the heat transfer coefficient of supercritical CO_2_ in horizontal straight tube, they found that the LB low Re turbulence model was better in predicting heat transfer, followed by standard *k*−*ε* model with enhanced wall treatment. In this paper the influence of wall thickness on the numerical simulation results is considered. So the model validation work is required.

**Fig 3:**
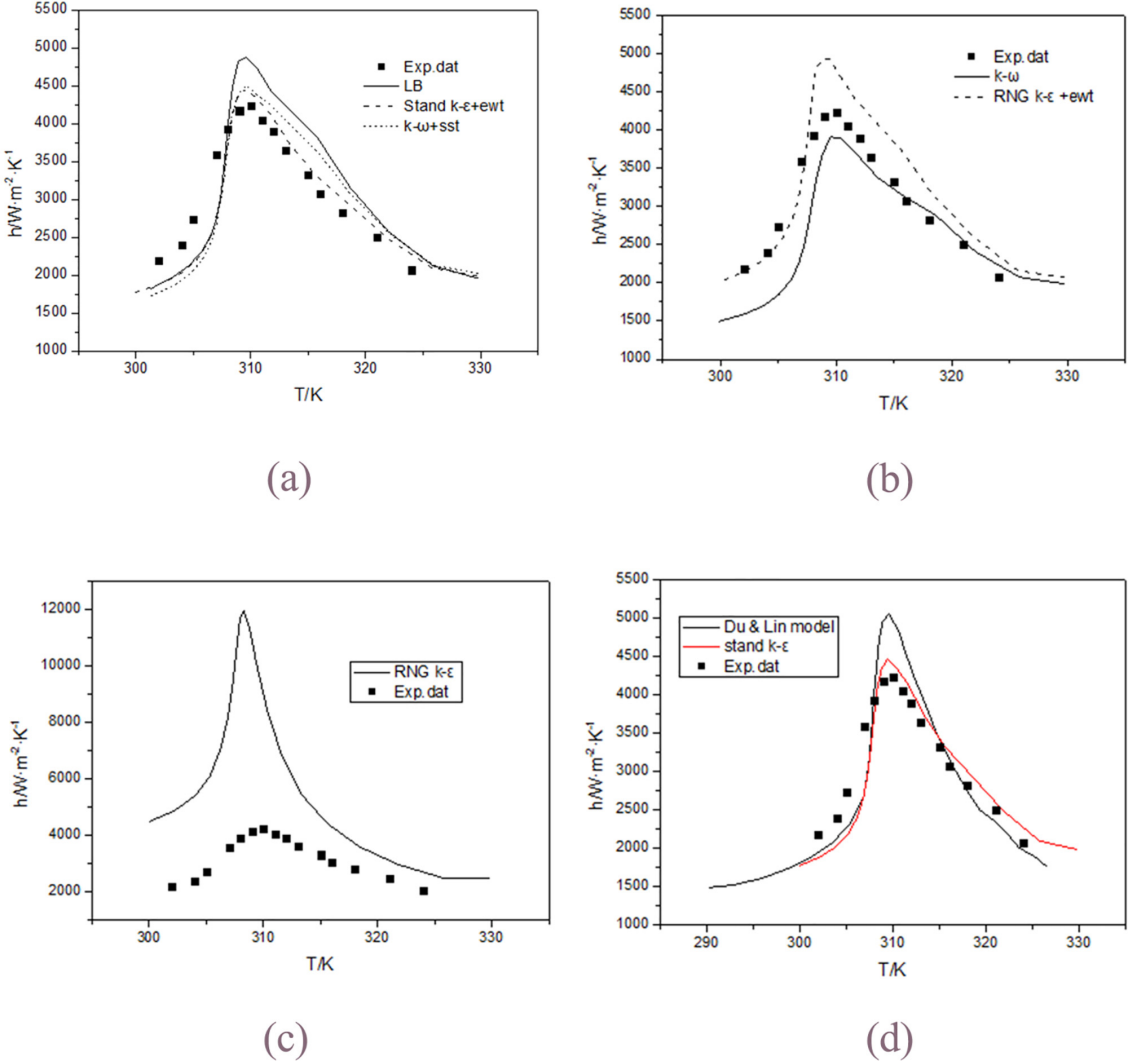
Comparisons of calculated heat transfer coefficient using various turbulence models with the experimental data of Dang and Hihara[4] at 33kW/m^2^. (a) LB, Stand k-ε+ ewt, k-ω +sst; (b) k-ω, RNG k-ε +ewt; (c) RNG k-ε; and (d) Stand k-ε, Du & Lin.dat.

Fig 3 shows the comparisons of calculated h with the experimental data. All turbulence models provide similar tendencies with the fluid temperature in the horizontal straight tube.

Fig 3(A) shows the calculated results of the standard *k*−*ε* with enhanced wall treatment model, the SST model, the LB model. The standard *k*−*ε* with enhanced wall treatment model well predicts the measured h except in the beginning. The LB model overestimates the heat transfer coefficient when T>308K and underestimates the heat transfer coefficient when T<308K, whereas the SST model slightly overestimates the heat transfer coefficient when T>308K and underestimates the heat transfer coefficient when T<308K.

Fig 3(B) shows the results of two turbulence models cannot predict the experimental h well. The *k*−*ω* model underestimates the experimental h when temperature is less than 316k. The RNG *k*−*ε* with enhanced wall treatment model overestimates the experimental h when temperature is greater than 307k.

Fig 3(C) shows the calculated results of the RNG *k*−*ε* model. The model seriously overestimates the heat transfer coefficient in all the regions.

From the discussion above, among all the turbulence models, the standard *k*−*ε* with enhanced wall treatment model gives the best prediction to the experimental data. The maximum error between the experimental h and the predictions of this model is approaching to 20%.

Fig 3(D) shows a comparison among calculated h with the experimental data, the simulation values using the standard *k*−*ε* with enhanced wall treatment model and the simulation values by Du and Lin[5]. The present results are better than that of Du and Lin. Considering the parameter setting differences in the numerical simulation, it can be considered that this simulation values are in good agreement with the experimental values and reflect the heat transfer characteristics of the flow. So the correctness of the numerical model and the parameter setting are proved.

In the paper another experimental model is needed to use. The experimental data used get from the experiment of Liu and He[7]. In the experiment, supercritical CO_2_ was cooled by cooling water. The test section was a tube-in-tube heat exchanger with 1300mm long, and the simulated conditions performed are that: mass flux is 92kg/m^2^s, the inlet pressure is at 8MPa, the inner diameter is 10.7mm, the inlet temperature is at 330 K, while heat flux is 26 kWm^-2^.

**Fig 4:**
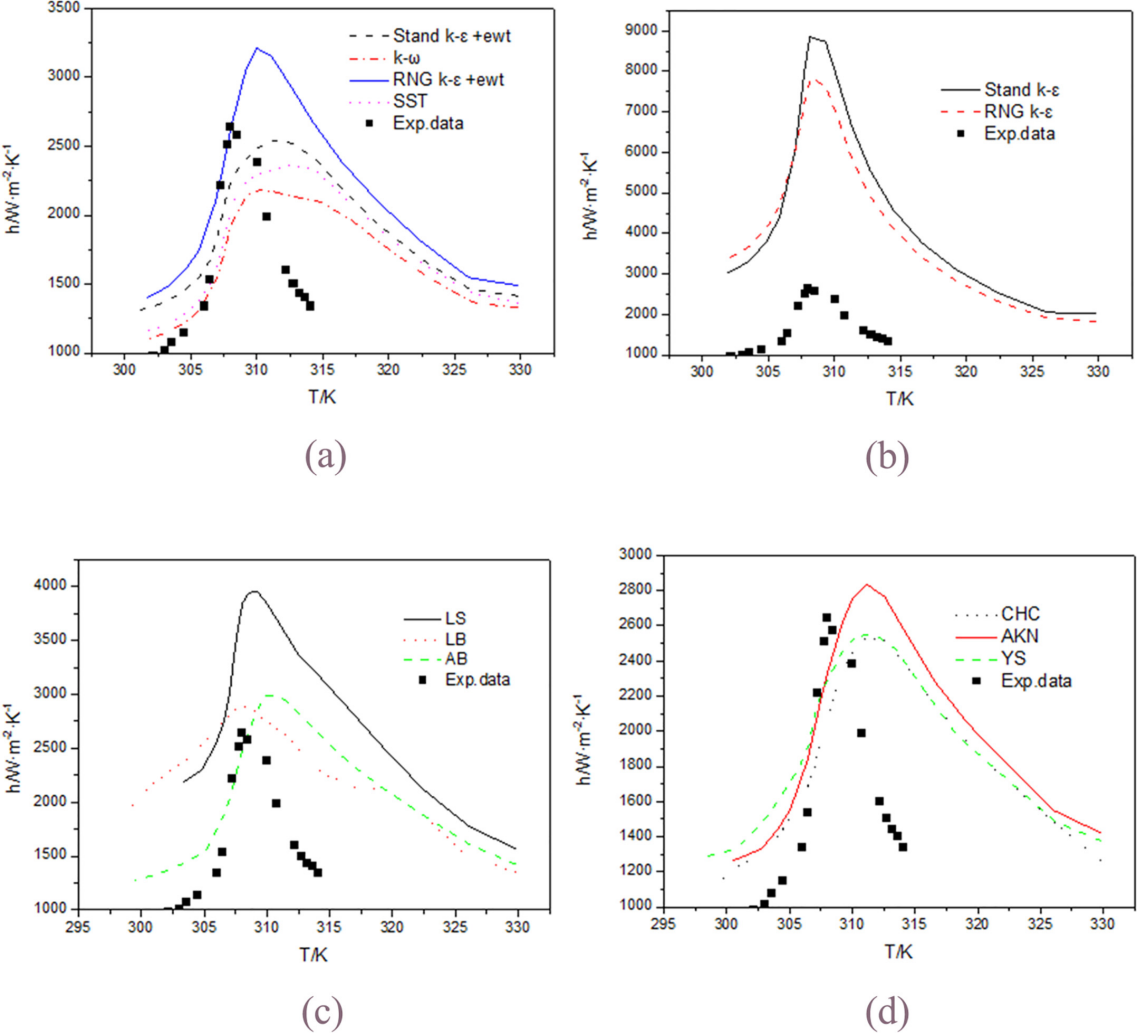
Comparisons of calculated heat transfer coefficient using various turbulence models with the experimental data of Liu and He. (a)Stand k-ε +ewt, k-ω, RNG k-ε +ewt, SST; (b) Stand k-ε, RNG k-ε; (c) AB, LB, LS; and (d) YS, AKN, CHC.

As mentioned in mathematical models, thirteen models are selected to simulate cooling heat transfer of supercritical CO_2_. In order to get a contrast between numerical prediction and the experimental data, the thirteen models are divided into four groups. Fig 4A–4D show the comparisons of calculated heat transfer coefficient using various turbulence models with the experimental data of Liu and He. It can be seen that almost all turbulence models are able to present the trend of heat transfer characteristics of supercritical CO_2_ cooling qualitatively. Although the inlet temperature and heat flux are different with the experiment of Liu and He, the peak of the heat transfer coefficient is often regarded as the most important aspect to examine the reliability of the models, so standard k–ε model with enhanced wall treatment is more accurate to predict heat transfer coefficient than other models, followed by LB low Re turbulence model.

According to the above studying, it can be concluded that the values simulated by the stand *k*−*ε* and RNG *k*−*ε* model are far from the experimental values for the horizontal straight tube with wall thickness, and the stand *k*−*ε* with enhanced wall treatment model gives the best prediction to the experimental data, followed by LB low Re turbulence model.

### 3.2. Velocity and turbulence distributions

**Fig 5:**
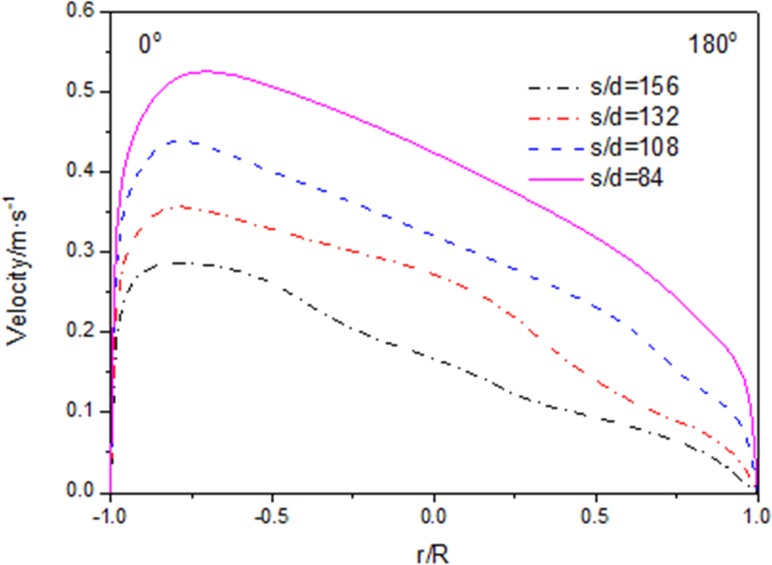
Radial distributions of velocity magnitude.

**Fig 6:**
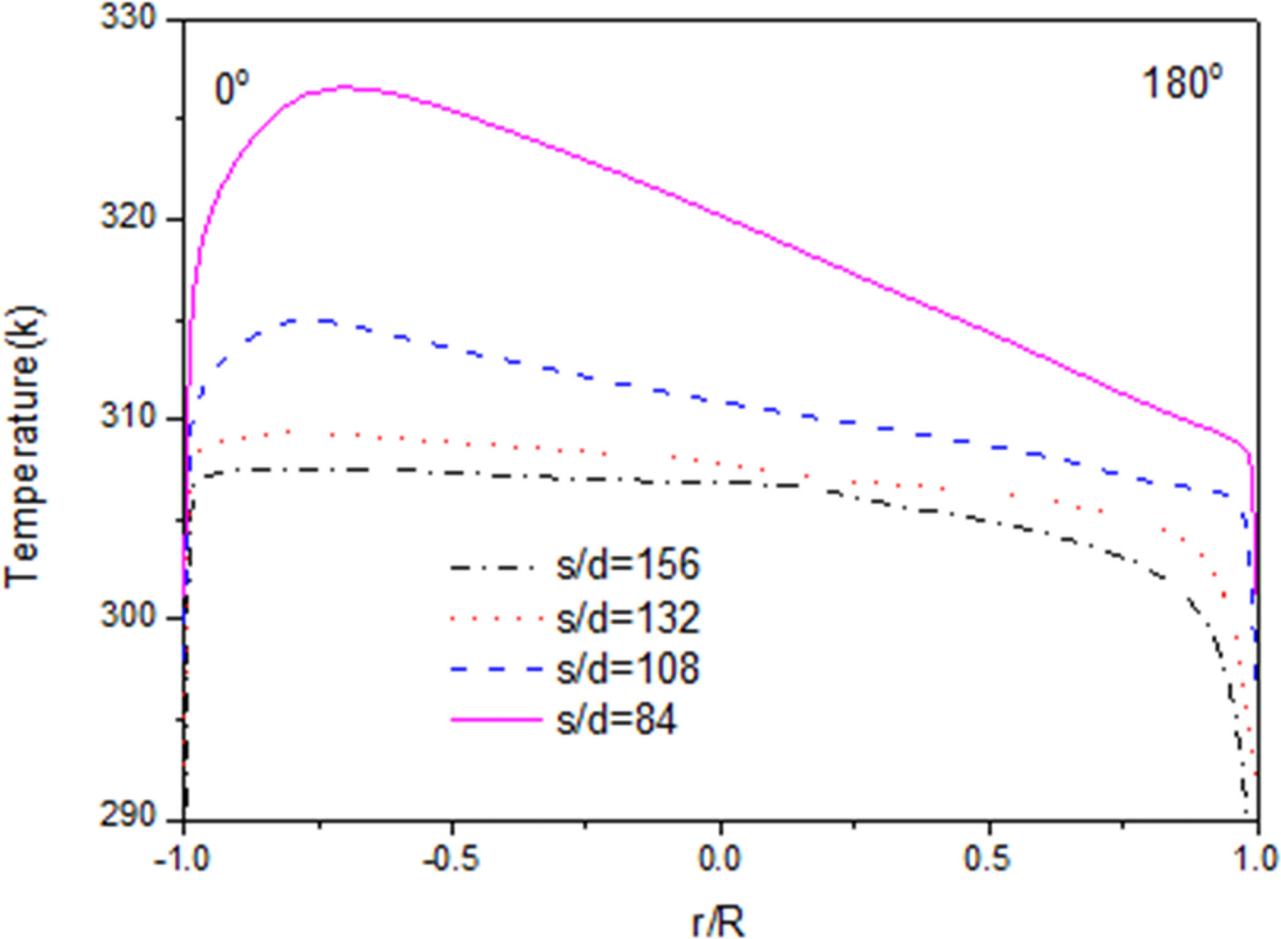
Radial distributions of fluid temperature.

**Fig 7:**
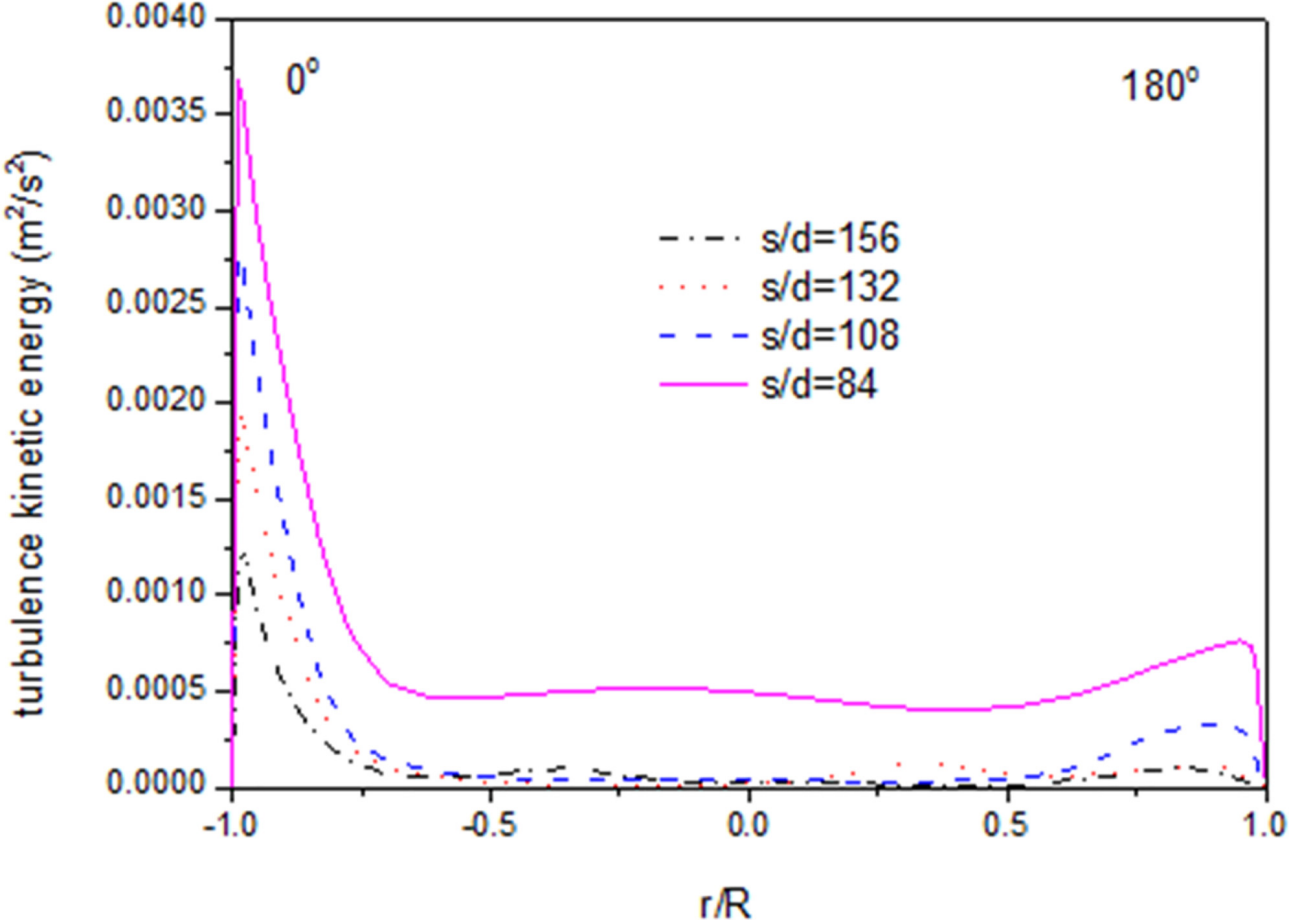
Radial distributions of turbulence kinetic energy.

Figs 5–7 show the distributions of velocity, fluid temperature and turbulence kinetic energy at different axial locations along the flow direction when the heat flux is 26kW/m^2^. It can be seen that velocity and turbulence kinetic energy gradually decrease with fluid temperature decreasing along the flow direction. The distributions of velocity, fluid temperature and turbulence kinetic energy are not axis-symmetric. The maximal gradient of the velocity is consistent with the maximal fluid temperature and the minimal turbulence kinetic energy exactly. The maximal velocity, maximal fluid temperature and the minimal turbulence kinetic energy locate at the position of r/R = -0.75, which clarify that fluid temperature of the top zone is higher than that of the bottom zone. The buoyancy effect is principally responsible for the asymmetry of velocity and temperature values at various axial locations. Because the CO_2_ with high temperature will go up to the top zone, and the CO_2_ with low temperature will go down to the bottom zone under the buoyancy effect.

### 3.3. The criterion of buoyancy effect on convection heat transfer

Buoyancy effect is an important issue in the study of cooling heat transfer to supercritical CO_2_. For a long time, there are several non dimensional numbers, which are used to determine the effect of buoyancy on the convective heat transfer.

The Prandtl number reflects the contrast between the fluid momentum diffusion ability and energy diffusion ability. When P<1, the thickness of velocity boundary layer is less than that of the temperature boundary layer, when P>1, the velocity boundary layer thickness is larger than the temperature boundary layer thickness. Because the influence of viscosity is bigger, the thickness of the flow boundary layer and the temperature boundary layer are thicker.

The Richardson number Ri is defined as the ratio of the buoyancy to the inertial forces. Ri = *Gr*/Re^2^ is always used to estimate the impact of buoyancy. Generally, when *Gr*/Re^2^ ≥ 0.01, the natural convection effect caused by gravity should be considered and the buoyancy will significantly influence the heat transfer; when *Gr*/Re^2^ ≥ 10 the natural convection takes effect and the forced convection can be neglected. When 0.1≤*Gr*/Re^2^≤10 the mixed convection is taking effect, and the two effects should be taken into consideration.

The term *Gr*/Re^2.7^ proposed by Jackson et al.[16] is also used to represent the effect of buoyancy. The criterion proposes that buoyancy effect cannot neglect when

Gr/Re2.7>10−5.

In order to estimate the effect of the buoyancy force on the heat transfer, the dimensionless buoyancy number Bo introduced by Jackson et al. [17] is adopted, which is defined as

Bo=GrRe3.425Pr0.8 (1)

This number represents the ratio of the buoyancy force to the inertial force. McEligot and Jackson [18] believed that when the buoyancy number Bo > 6 x10^-7^, the buoyancy effect was strong enough to cause the mixed convection.

McEligot et al.[19] investigated the influence of flow acceleration due to heating. McEligot et al. introduced a acceleration parameter to describe the effect of flow acceleration due to heating on the heat transfer:

Kv=4qwdRe2μcpT (2)

McEligot et al. suggested that when *K*_*V*_ ≤ 3×10^−6^ the fluid flow remained turbulent. When *K*_*V*_ ≥ 3×10^−6^ the turbulence might be significantly reduced and the flow may even re-laminarized, which reduced the overall heat transfer.

### 3.4. Wall effect and heat flux effect

**Fig 8:**
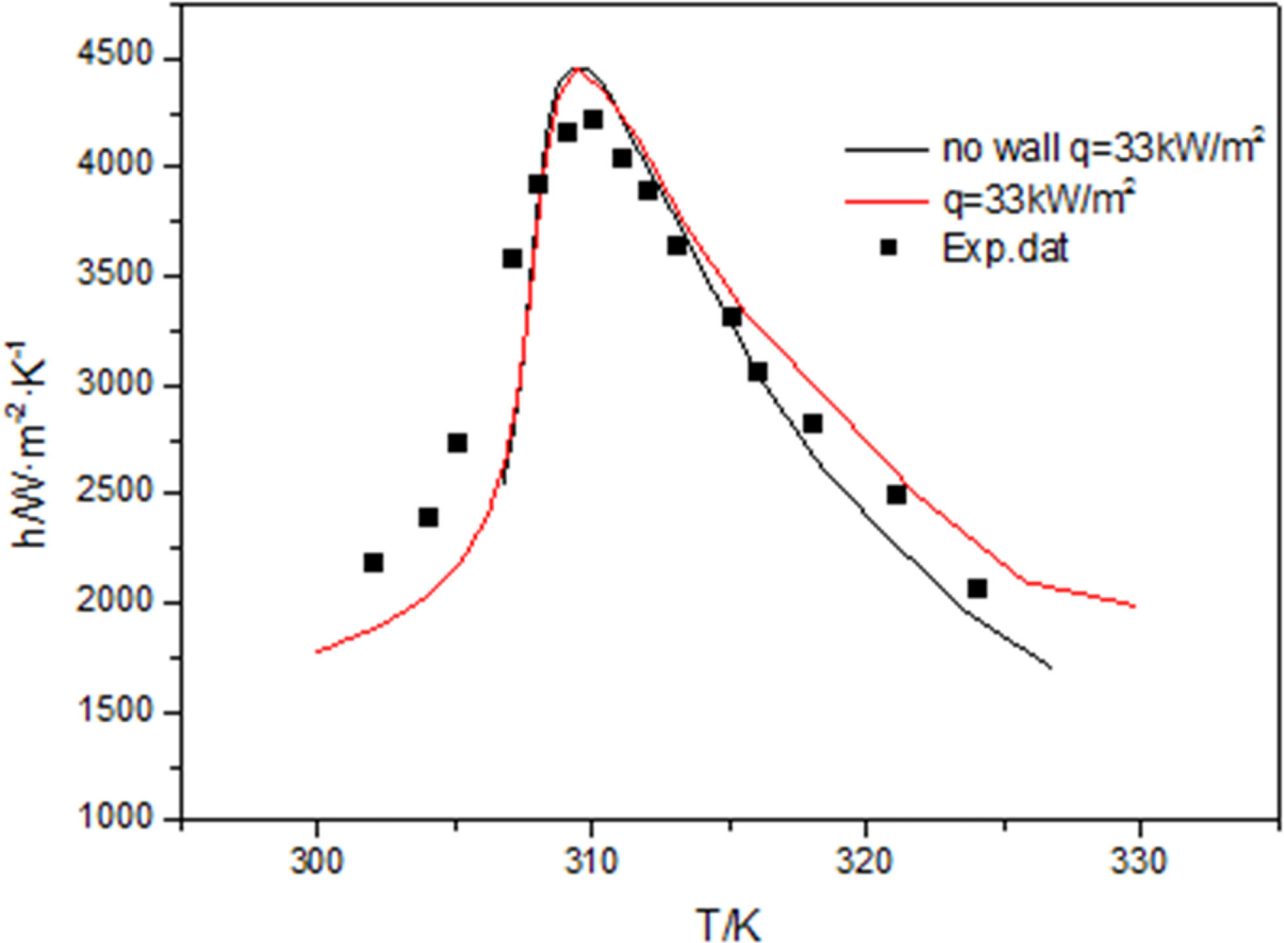
Comparisons of heat transfer coefficient with wall and without wall at 33kW/m^2^.

Fig 8 compares the numerical results with and without the wall with the experimental data. The simulated conditions performed are that mass flux is 200 kg/m^2^, the inlet pressure at 8MPa, the inlet temperature at 330 K, the heat flux at 33 kW/m^2^.The results show that the numerical values with the wall predict more closely the experimental data than those without the wall when T>315K. Because at the beginning the heat flux on the tube wall conducts the heat through the tube wall, when T<315K, the tube wall temperature is stable and the heat transfer coefficient with wall is exactly the same with that without wall.

**Fig 9:**
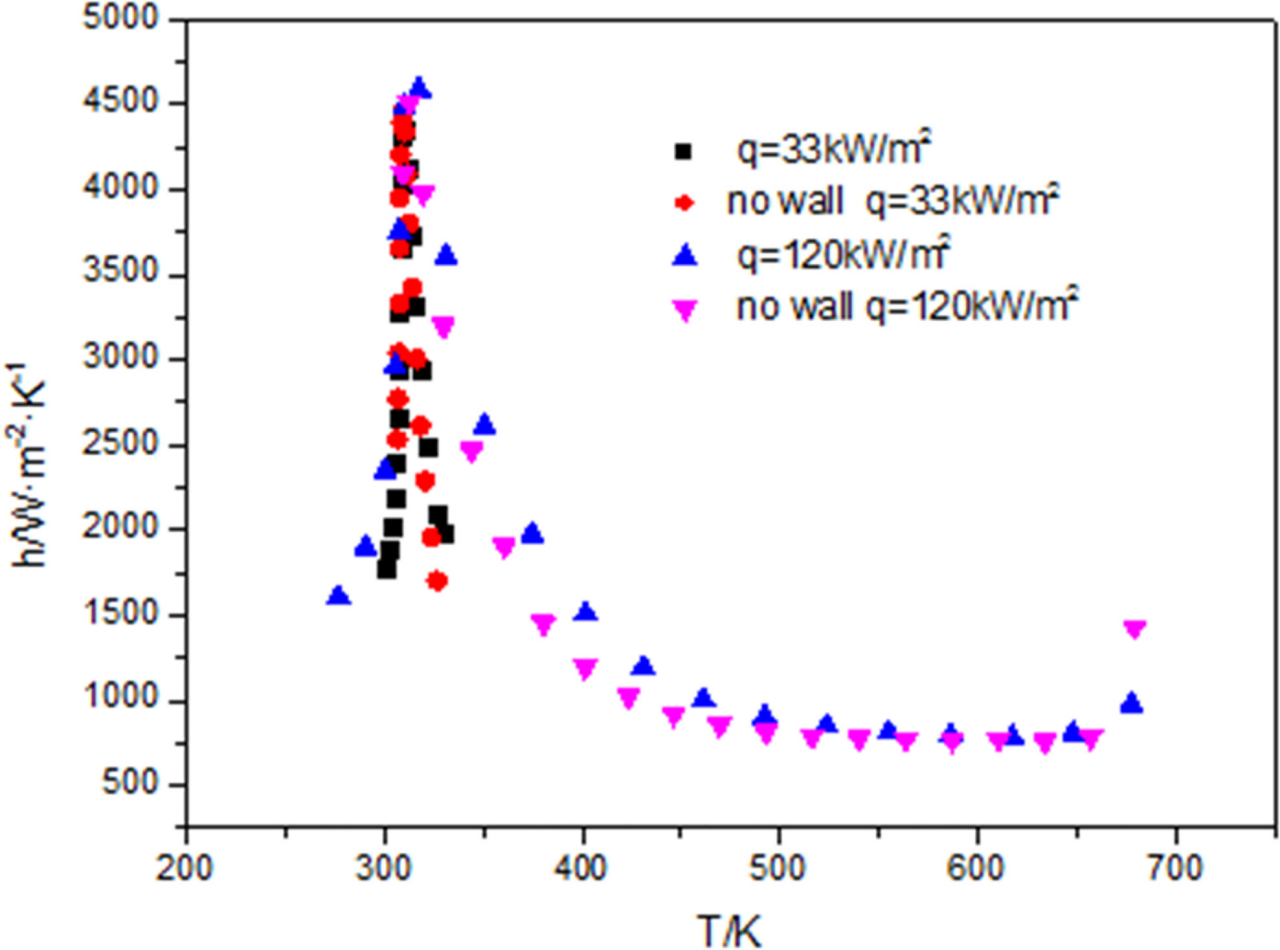
Comparisons of heat transfer coefficient with wall and without wall at 33kW/m^2^ and 120kW/m^2^ with the fluid temperature.

Fig 9 shows before the critical temperature, heat transfer coefficients are the same with wall and without the wall whether the heat flux is 33kW/m^2^ or 120kW/m^2^. After the critical temperature, the heat transfer coefficient with wall is greater than that without the wall.

**Fig 10:**
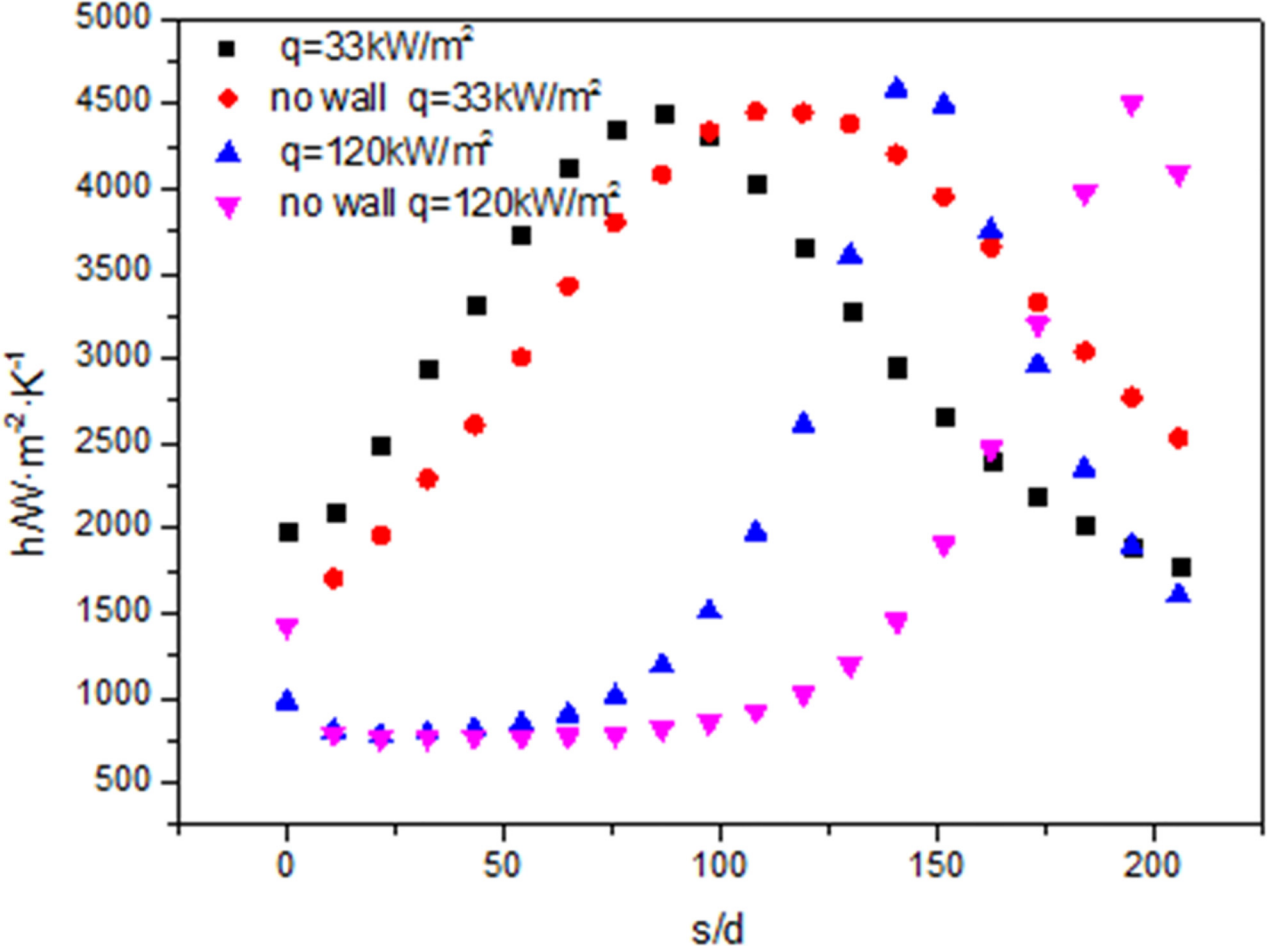
Comparisons of heat transfer coefficient with wall and without wall at 33kW/m^2^ and 120kW/m^2^ along the tube.

Fig 10 shows when the heat flux is 33 kW/m^2^, the heat transfer coefficient reaches the maximum value at s/d = 86 with wall, and the heat transfer coefficient reaches the maximum value at s/d = 108 without the wall. When the heat flux is 120 kW/m^2^, the heat transfer coefficient reaches the maximum value at s/d = 140 with wall and the heat transfer coefficient reaches the maximum value at s/d = 195 without the wall. The fluid with wall is earlier than that without wall at reaching the maximum value.

**Fig 11:**
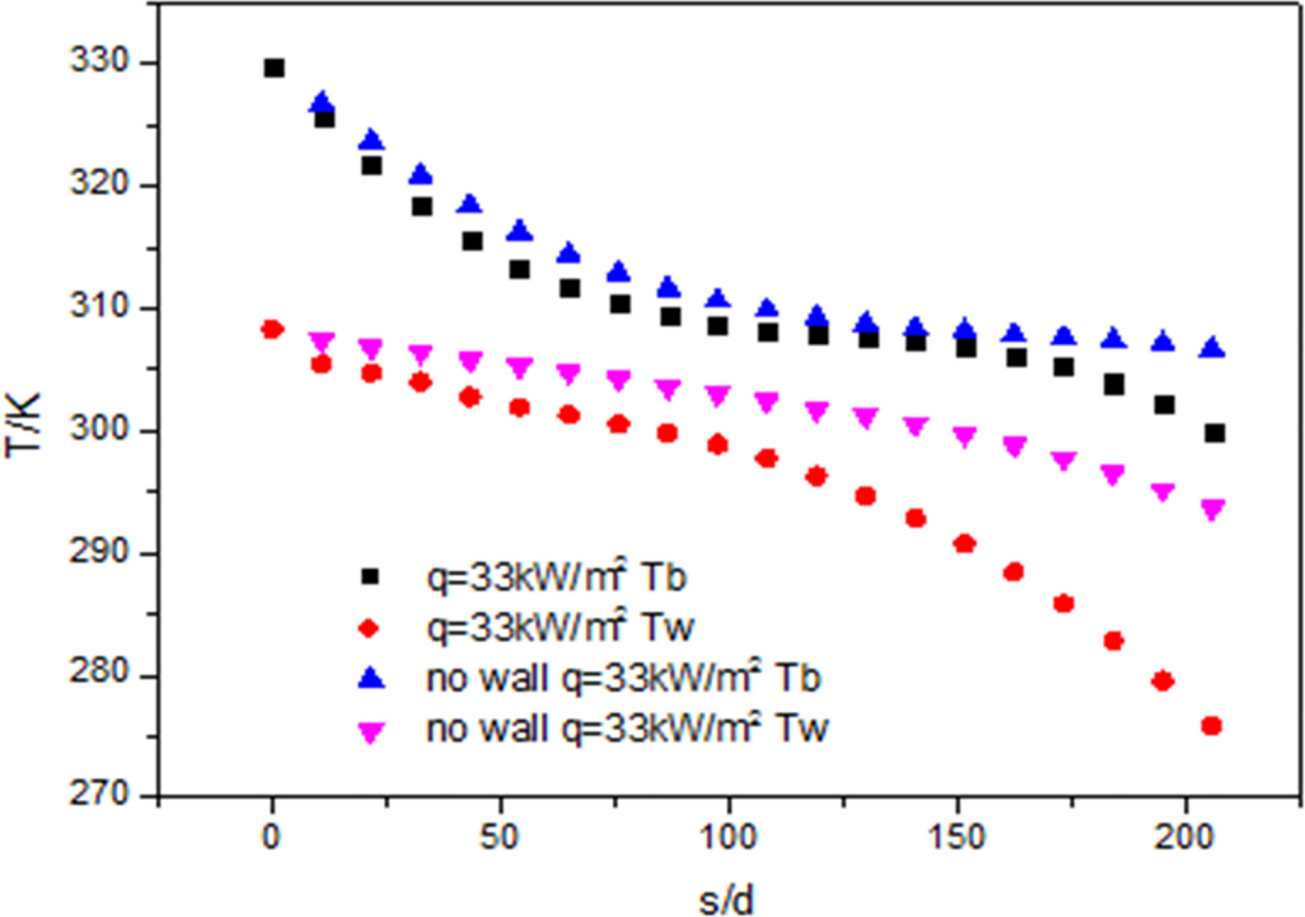
Development of wall temperature (Tw) and flow temperature (Tf) at 33kW/m^2^

Fig 11 shows the trends of wall temperature and fluid temperature when q = 33kW/m^2^. The wall temperature and fluid temperature with the wall are lower than that without the wall. Along the flow direction of the fluid, the fluid temperature difference between with wall and without the wall is small. The wall temperature difference between with wall and without the wall gradually increases.

**Fig 12:**
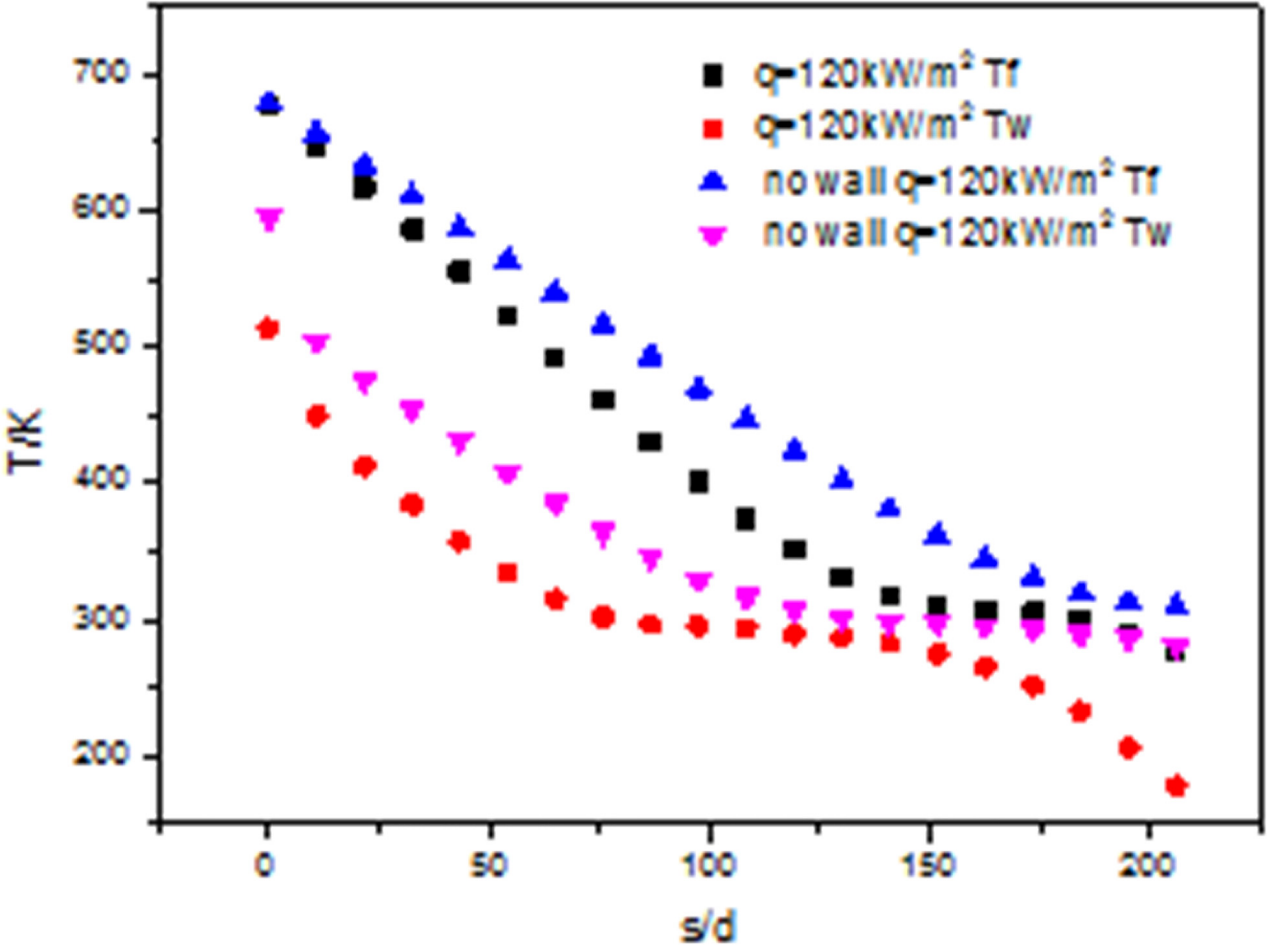
Development of wall temperature (Tw) and flow temperature (Tf) at 120kW/m^2^.

Fig 12 shows the trends of wall temperature and fluid temperature when q = 120kW/m^2^. The wall temperature and fluid temperature with the wall are lower than that without the wall. Along the direction of the fluid flow, the fluid temperature difference and the wall temperature difference between with wall and without the wall are larger and the differences get the smallest near the critical temperature.

### 3.5. The small heat flux effect

As shown in Fig 9 above, the maximum heat transfer coefficient is not changed when the heat flux increases from 33kW/m^2^ to 120kW/m^2^, the temperature at which the maximum heat transfer coefficient happens still has not changed with the fluid temperature, and is also at the position of the critical temperature. The cooling temperature range of CO_2_ increases from 330K to 680K when the heat flux increases from 33kW/m^2^ to 120kW/m^2^.

As shown in Fig 10, when the heat flux is 33 kW/m^2^, the heat transfer coefficient reaches the maximum value at s/d = 86; when the heat flux is 120 kW/m^2^, the heat transfer coefficient reaches the maximum value at s/d = 140. The fluid with lower heat flux is earlier than that with higher heat flux at reaching the maximum value. Because the maximum value of the heat transfer coefficient is certain to occur in the vicinity of the critical temperature. When the heat flux is 33kW/m^2^, the fluid temperature reaches critical temperature at s/d = 75, and when the heat flux is 120kW/m^2^, the fluid temperature reaches critical temperature at s/d = 150, so the maximum value of the heat transfer coefficient at 33kW/m^2^ happens earlier than that at 120kW/m^2^.

From the above Figs 11 and 12, it can be concluded that the fluid and the wall temperature range are different. When the heat flux is 120kW/m^2^, the temperature range is from 177K to 677K. When the heat flux is 33kW/m^2^, the temperature range is from 275K to 329K. The former is bigger than the later. The maximal difference of the wall and fluid temperature with and without wall at 120kW/m^2^ is 205K. The maximal difference of the wall and fluid temperature with and without wall at 33kW/m^2^ is 24K. The former is about eight times as much as the latter.

**Fig 13:**
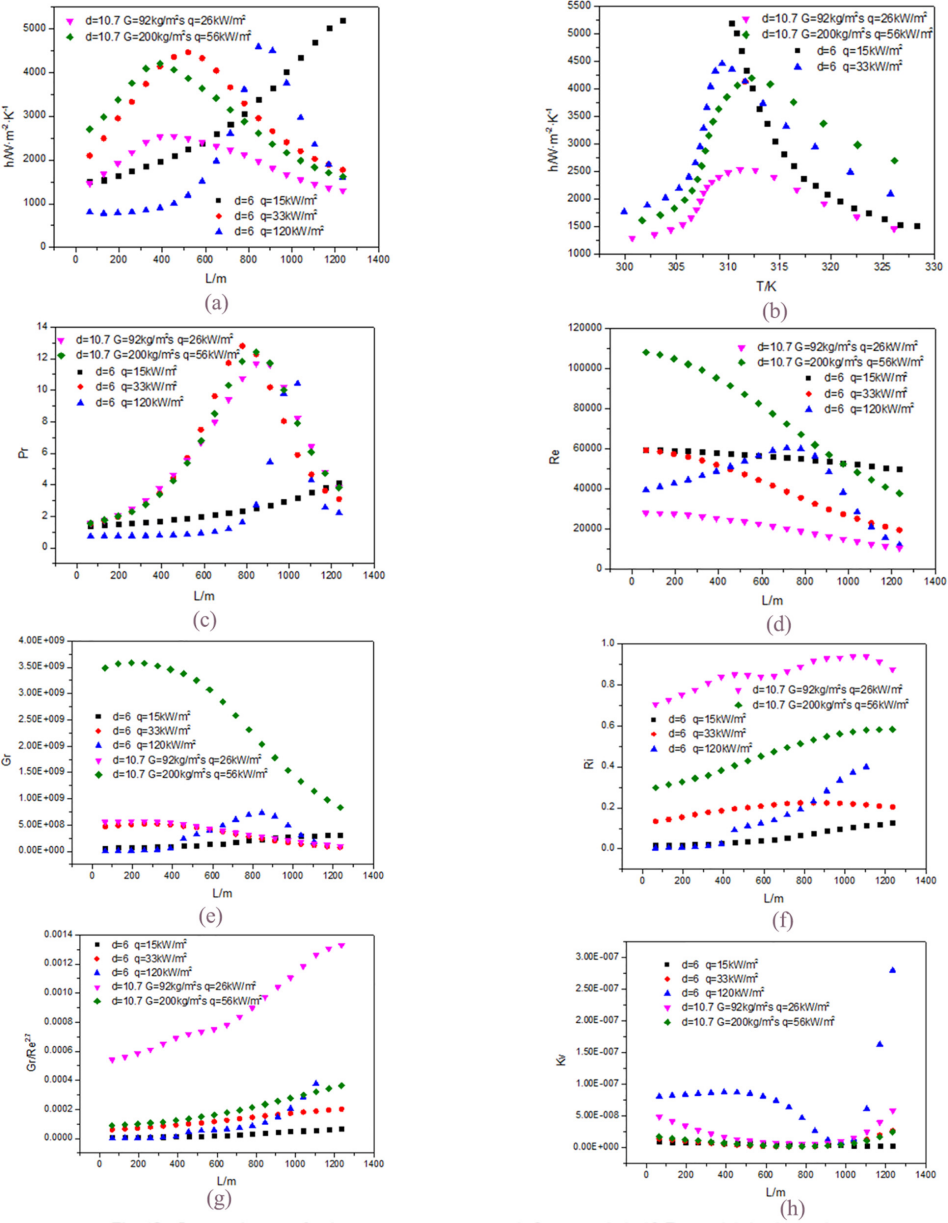
Comparisons of relevant parameters at d = 6mm and d = 10.7mm.(a) h along the tube; (b) h with the fluid temperature; (c) Pr; (d) Re; (e) Gr; (f) Ri; (g) Gr/Re^2.7^; (h) Kv.

These are in accordance with that shown in Fig 13A and 13B. This fully shows that the change of heat flux does not affect maximum value of the heat transfer when the diameter and the mass flow rate are the same and the heat flux will only increase the cooling temperature.

As Fig 13(C) shown the maximum value of Pr at lower heat flux is bigger than that at higher heat flux. In reaching the maximum, the speed of former is faster than that of the later. These are consistent with the change trend of the heat transfer coefficient.

As Fig 13D and 13E shown, the curve Re and Gr at 33kW/m^2^ decrease gradually along the flow direction. Because the fluid velocity decreases, viscosity increases and density increases with the CO_2_ temperature decrease.

As Fig 13E and 13E shown, the curve Re and Gr at 120kW/m^2^ first increase and then decrease gradually along the flow direction. Due to the large heat flux, the range of cooling temperature is larger, and the density increases in wide range. At the same time the fluid velocity decreases, viscosity increases with the CO_2_ temperature decrease. Based on the synthesis of the above reasons, Gr and Re firstly increase and then decrease.

Though both Re and Gr decrease, the impact of Re is greater than that of Gr. So the Ri increases as the Fig 13(F) shown. When the heat flux is 33 kW/m^2^, the change trend of Ri is relatively flat. When the heat flux is 120 kW/m^2^, the change trend of Ri is larger.

At the same time the increase in Gr/RE^2.7^ is greater than that in Ri as shown in Fig 13(G). When the heat flux is 33kW/m^2^, the change trend of Gr/RE^2.7^ is relatively flat, and when the heat flux is 120kW/m^2^, the change trend of Gr/RE^2.7^ is larger, because the Re at 33kW/m^2^ is greater than that at 120kW/m^2^.

Under the influence of various parameters, the curve of Kv at 33 kW/m^2^ and at 120 kW/m^2^ first decreases and then increases, and the variation range at 120 kW/m^2^ is larger than that at 33 kW/m^2^ as shown in Fig 13(H).

When the heat flux is 15 kW/m^2^, the fluid in the tube does not reach the maximum heat transfer coefficient, although the heat flux is small, the difference between the wall temperature and fluid temperature is also small, so the heat transfer coefficient when heat flux is 15 kW/m^2^ is bigger than that when heat flux is 33 kW/m^2^. The change trends of other parameters at 15kW/m^2^ are similar with that at 33kW/m^2^ as shown in Fig 13.

### 3.6. The mass flow rate effect

Fig 13(A) shows when the diameter is the same (d = 10.7mm), the heat flux and mass flow rate increase at the same proportion (G_1_ = 92kg/m^2^s, q_1_ = 26kW/m^2^, G_2_ = 200kg/m^2^s, q_1_ = 56kW/m^2^), the heat transfer coefficient achieves the maximum value at the same position along the flow direction. The fluid temperatures under 26kW/m^2^ and 56kW/m^2^ at the same position along the flow direction are the same. For the wall temperature under 56kW/m^2^ is smaller than that under 26kW/m^2^ at the same position along the flow direction. When the heat flux is 56kW/m^2^, the heat transfer coefficient is bigger than that when the heat flux is 26kW/m^2^ but the ratio is less than two times as shown in Fig 13(B).

Fig 13(C) shows the Pr values reach the maximum in the same position at 26kW/m^2^ and 56kW/m^2^ that is in keeping with change condition of the heat transfer coefficient. The change trend of Re and Gr values are the same, they decrease gradually along the flow direction as shown in Fig 13D and 13E, because the fluid velocity decreases, viscosity increases and density increases with the CO_2_ temperature decrease. The values of Re and Gr with higher heat flux decrease more quickly than that with lower heat flux. It is because that the fluid temperatures under the two heat flux are the same along the flow direction, so the fluid viscosity and fluid density are the same too. But when the heat flux is 56kW/m^2^, the velocity is double of that when the heat flux is 26kW/m^2^ and the wall density at higher heat flux is higher than that at lower heat flux.

Though both Re and Gr decrease at the same time, the impact of Re is greater than that of Gr. So the Ri increases as shown in Fig 13(F). At the same time the increase in Gr/RE^2.7^ is greater than that in Ri as shown in Fig 13(G). Under the influence of various parameters, Kv first decreases, then increases as shown in Fig 13(H).

### 3.7. The large heat flux effect

**Fig 14:**
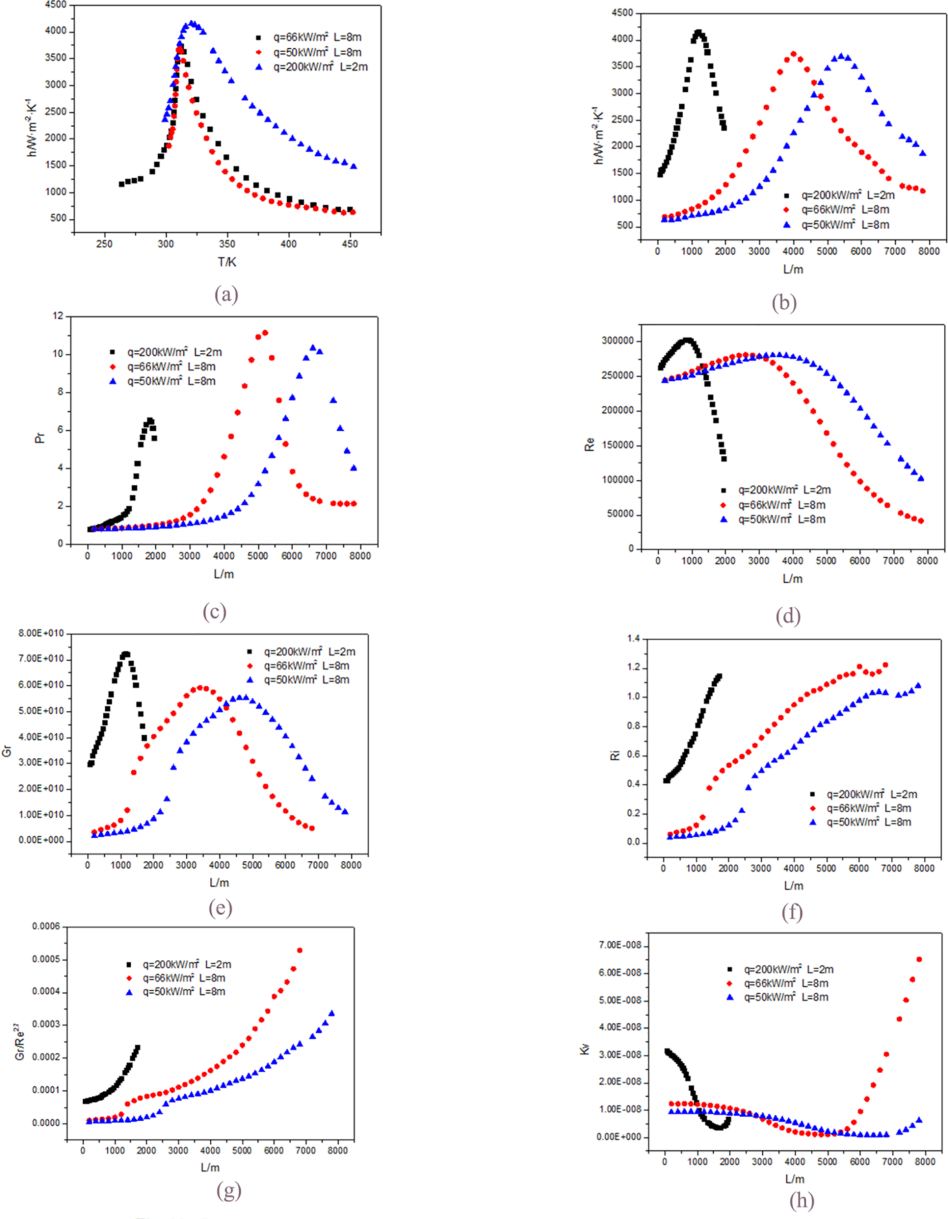
Comparisons of relevant parameters at d = 26mm. (a) h along the tube; (b) h with the fluid temperature; (c) Pr; (d) Re; (e) Gr; (f) Ri; (g) Gr/Re^2.7^; (h) Kv.

Fig 14(A) shows the influence of heat flux on the heat transfer coefficient of supercritical CO_2_ in horizontal straight tube under the same mass flux (G = 200kg/m^2^s), same diameter (d = 26mm) and same length (L = 8m). Before the critical temperature (like liquid zone), heat flux almost has no effect on the heat transfer coefficient; and after the critical temperature point (like gas zone), heat transfer coefficient slightly increases with the heat flux increase. This is because that in the like gas zone, specific heat capacity and thermal conductivity increase with the fluid temperature decrease, therefore, the heat transfer coefficient will increases with the heat flux increase; in the like liquid zone, specific heat capacity has been decreased with decrease of the fluid temperature but the thermal conductivity first decreases and then increases, so the heat flux almost has no effect on the heat transfer coefficient. It can be also concluded that at the same inlet temperature, when the heat flux is 66kW/m^2^, the outlet fluid temperature is 263K and the outlet wall temperature is 194K. When the heat flux is 50kW/m^2^, the outlet fluid temperature is 302K and the outlet wall temperature is 269K. The greater the heat flux is, the lower the temperature of the fluid is. This is in accord to the section 3.5. The heat flux will only increase the cooling temperature range.

Fig 14(B) shows when the heat flux is 50kW/m^2^ and the maximum value appears at L = 5400mm, when the heat flux is 66kW/m^2^, it appears at L = 4000mm and the later is earlier than the former. This is because when the heat flux is 50kW/m^2^, the fluid temperature reaches the critical temperature at L = 5600mm, and when the heat flux is 66kW/m^2^, the fluid temperature reaches critical temperature at L = 4200mm. So the maximum value of the heat transfer coefficient at 50kW/m^2^ happens later than that at 66kW/m^2^.

Fig 14(C) shows that the Pr value at 66kW/m^2^ is greater than that at 50kW/m^2^, and appears earlier than that at 50kW/m^2^. This change condition is consistent with that of the heat transfer coefficient. Fig 14(D) shows that the change trends of Re at 66 kW/m^2^ and at 50 kW/m^2^ are the same, the values first increases and then decreases along the length direction, and the increase range is small. It is result from the interaction of the fluid density and viscosity.

Fig 14(E) shows that the change trends of Gr at 66kW/m^2^ and at 50kW/m^2^ are the same too. Due to the large cooling temperature range from 460K to 263K at 66kW/m^2^, the fluid density changes in a wide range. Combined with the change of the fluid density and viscosity, the change trend of Gr is like a parabola.

Though both Re and Gr decrease, the impact of Re is greater than that of Gr. So the Ri increases just as the Fig 14(F) shown. At the same time the increase in Gr/RE^2.7^ is greater than that in Ri as shown in Fig 14(G). Under the influence of various parameters, Kv first decreases, then increases. The change scope at 66kW/m^2^ is greater than that at 50kW/m^2^ just as Fig 14(H) shown.

### 3.8. The tube length, inlet temperature and diameter effect

Fig 14 shows that when the mass flow rate (G = 200kg/m^2^s), the diameter (d = 26mm), the inlet and outlet fluid temperature are the same, the heat flux is proportional to the length of tube, the heat transfer coefficient at 200kW/m^2^ is higher than that at 50kW/m^2^. For the fluid temperatures under 200kW/m^2^ and 50kW/m^2^ at the same position along the flow direction are the same, the wall temperature under 200kW/m^2^ is lower than that under 50kW/m^2^ at the same position along the flow direction. But the impact of the temperature difference is smaller than that of heat flux. The change trends of other parameters at 200kW/m^2^ are similar with that at 50kW/m^2^.

**Fig 15:**
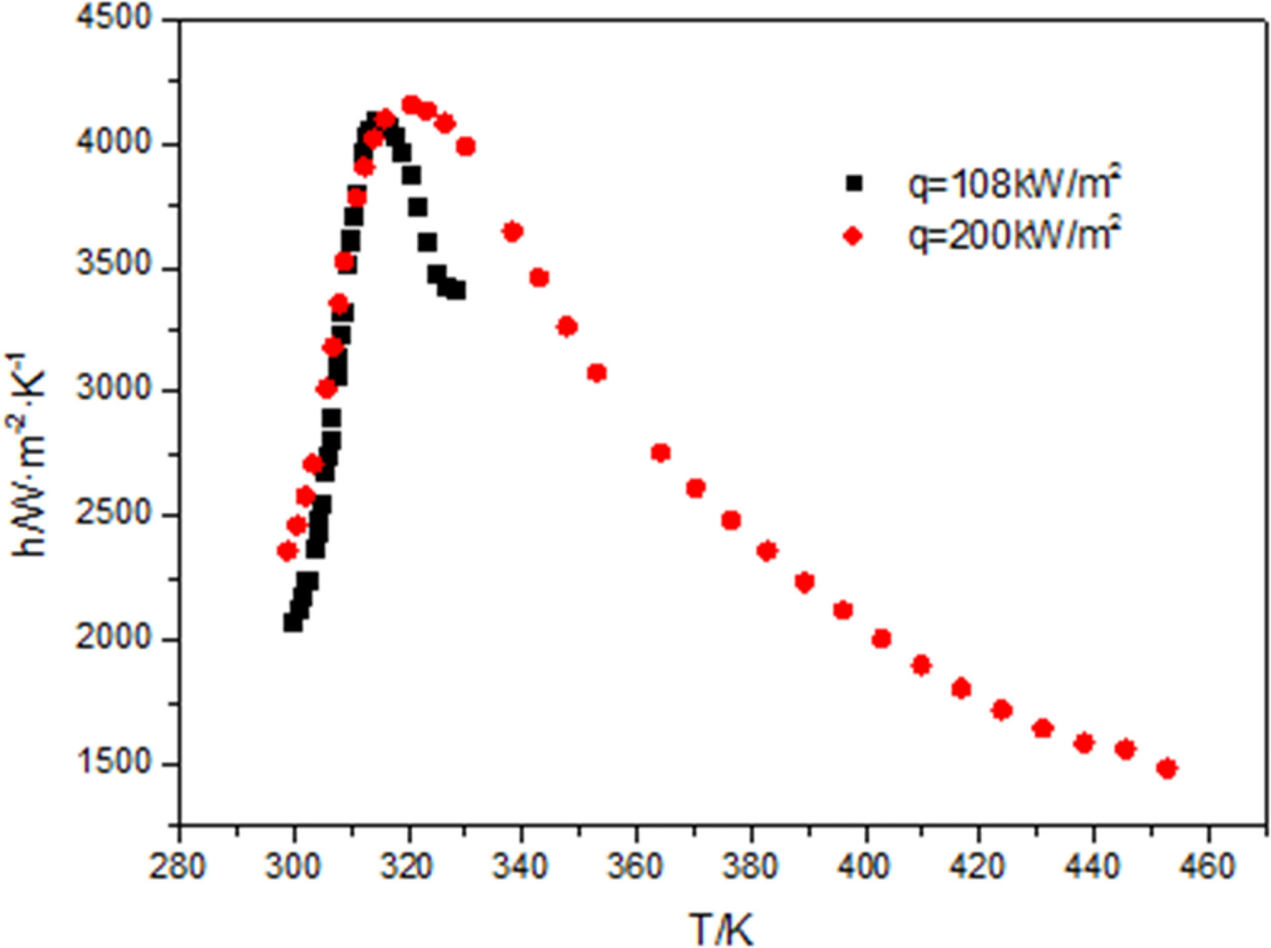
Comparisons of heat transfer coefficient at different inlet temperature with fluid temperature.

Fig 15 shows that when the mass flow rate (G = 200kg/m^2^s), the diameter (d = 26mm), and outlet fluid temperature are the same, the heat flux is proportional to the inlet fluid temperature, the maximum of heat transfer coefficient at 200kW/m^2^ is the same with that at 108kW/m^2^. The fluid temperatures and wall temperatures under 200kW/m^2^ and 108kW/m^2^ at the same position along the flow direction are not the same. Under the combined action of the wall temperature, fluid temperature, and heat flux, the maximum of heat transfer coefficient is the same.

**Fig 16:**
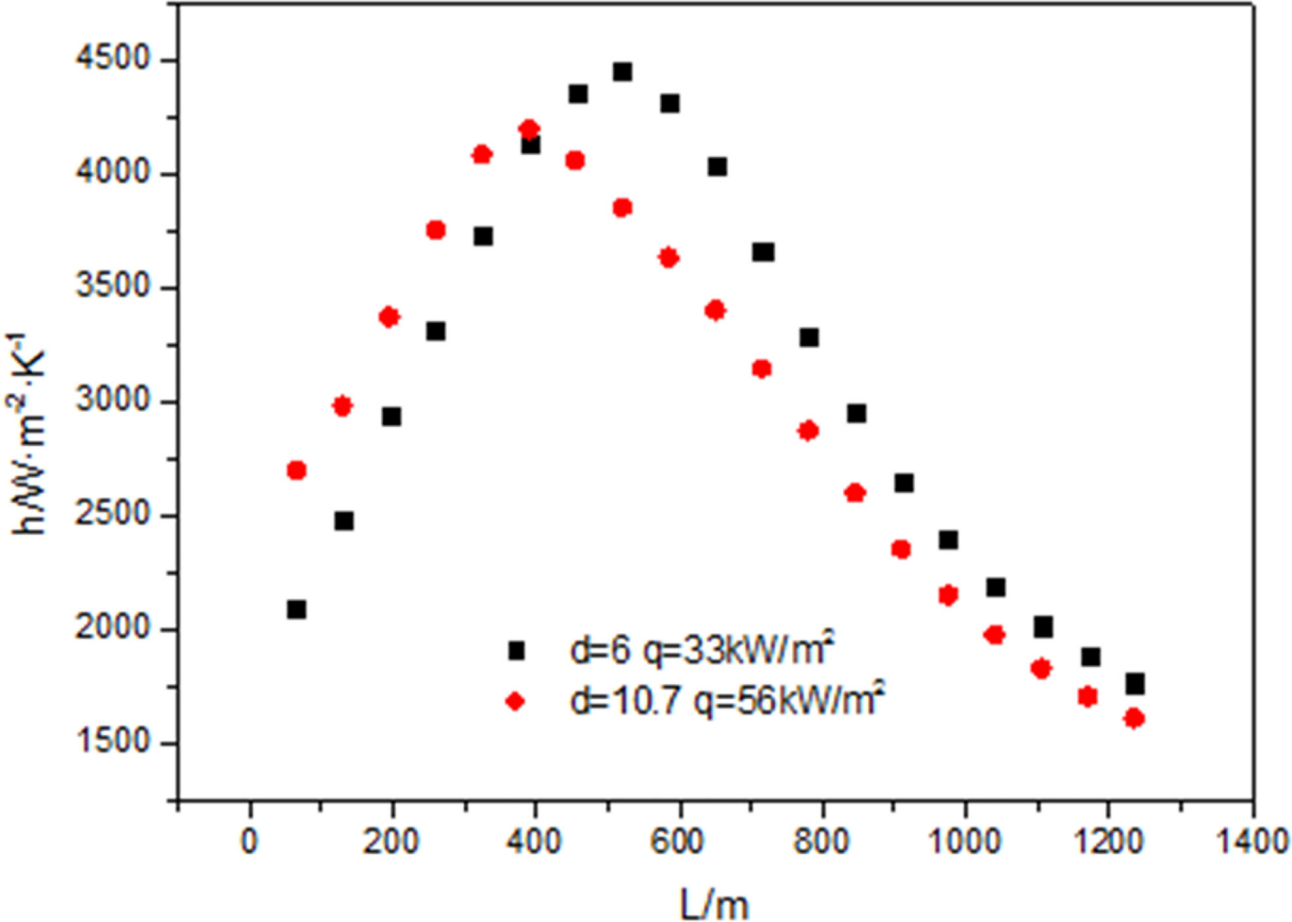
Comparisons of heat transfer coefficient at different diameter along the tube.

**Fig 17:**
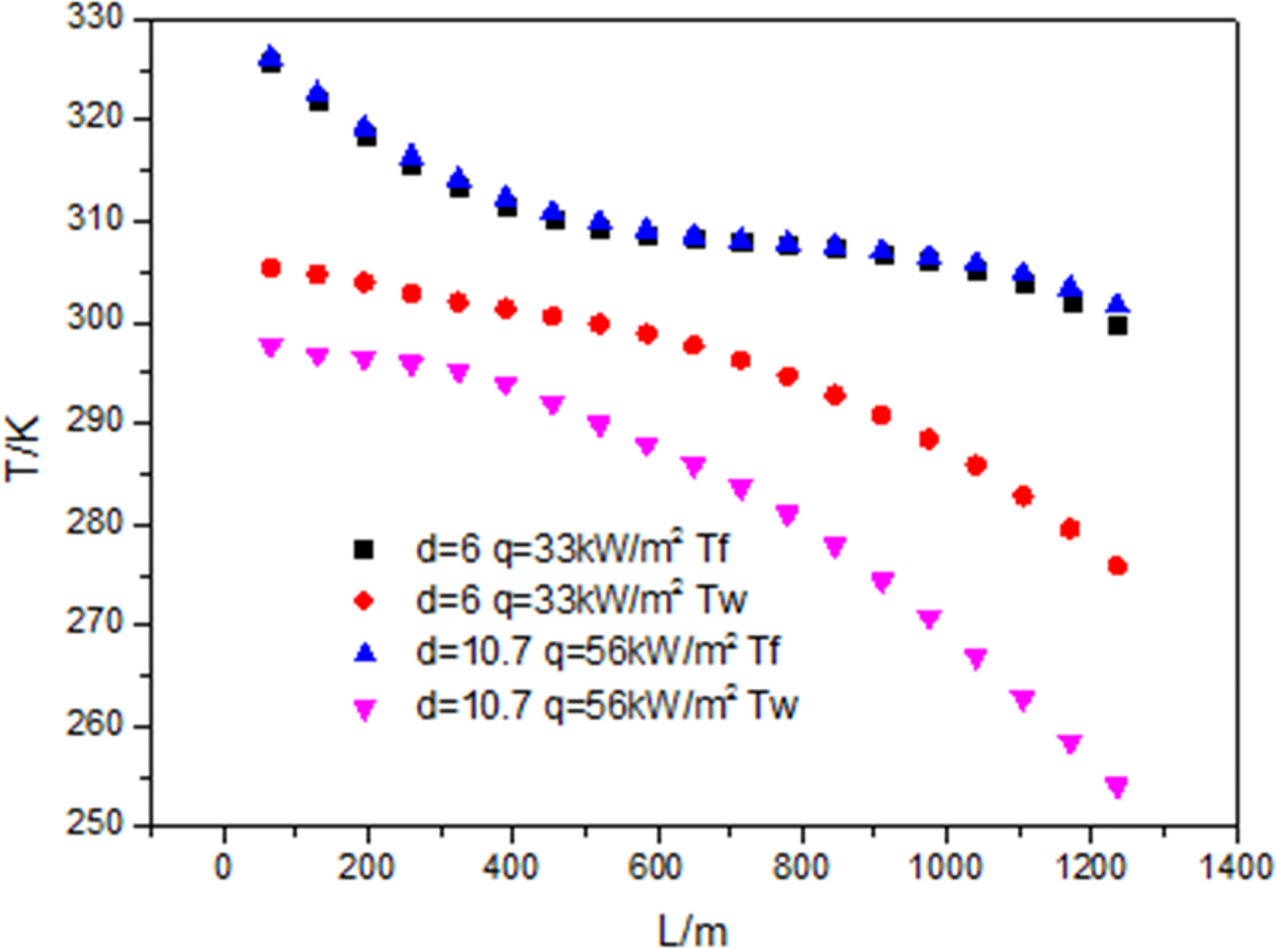
Development of wall temperature (Tw) and flow temperature (Tf) at different diameter.

Fig 16 shows that when the mass flow rate (G = 200kg/m^2^s), the inlet and outlet fluid temperature are the same, the heat flux is proportional to the diameter of tube (d = 6mm, d = 10.7mm), the heat transfer coefficient at 33kW/m^2^ is higher than that at 56kW/m^2^. For the fluid temperatures under 33kW/m^2^ and 56kW/m^2^ at the same position along the flow direction are the same, the wall temperature under 56kW/m^2^ is lower than that under 33kW/m^2^ at the same position along the flow direction just as shown in Fig 17. The impact of the temperature difference is a little bigger than that of heat flux. When the heat flux is 33kW/m^2^ and 56kW/m^2^, the fluid temperature reaches critical temperature at L = 455mm, but the distribution of the wall temperature is different, so the maximum value of the heat transfer coefficient at 56kW/m^2^ happens earlier than that at 33kW/m^2^.

### 3.9. Comparison between the heating and cooling condition

In order to study the difference between heating and cooling of the CO_2_ in the straight tube, the change of heat transfer coefficient on heating under the same length, diameter, and the mass flow rate is compared and the results demonstrate that heat transfer coefficient on heating and cooling is not the same.

**Fig 18:**
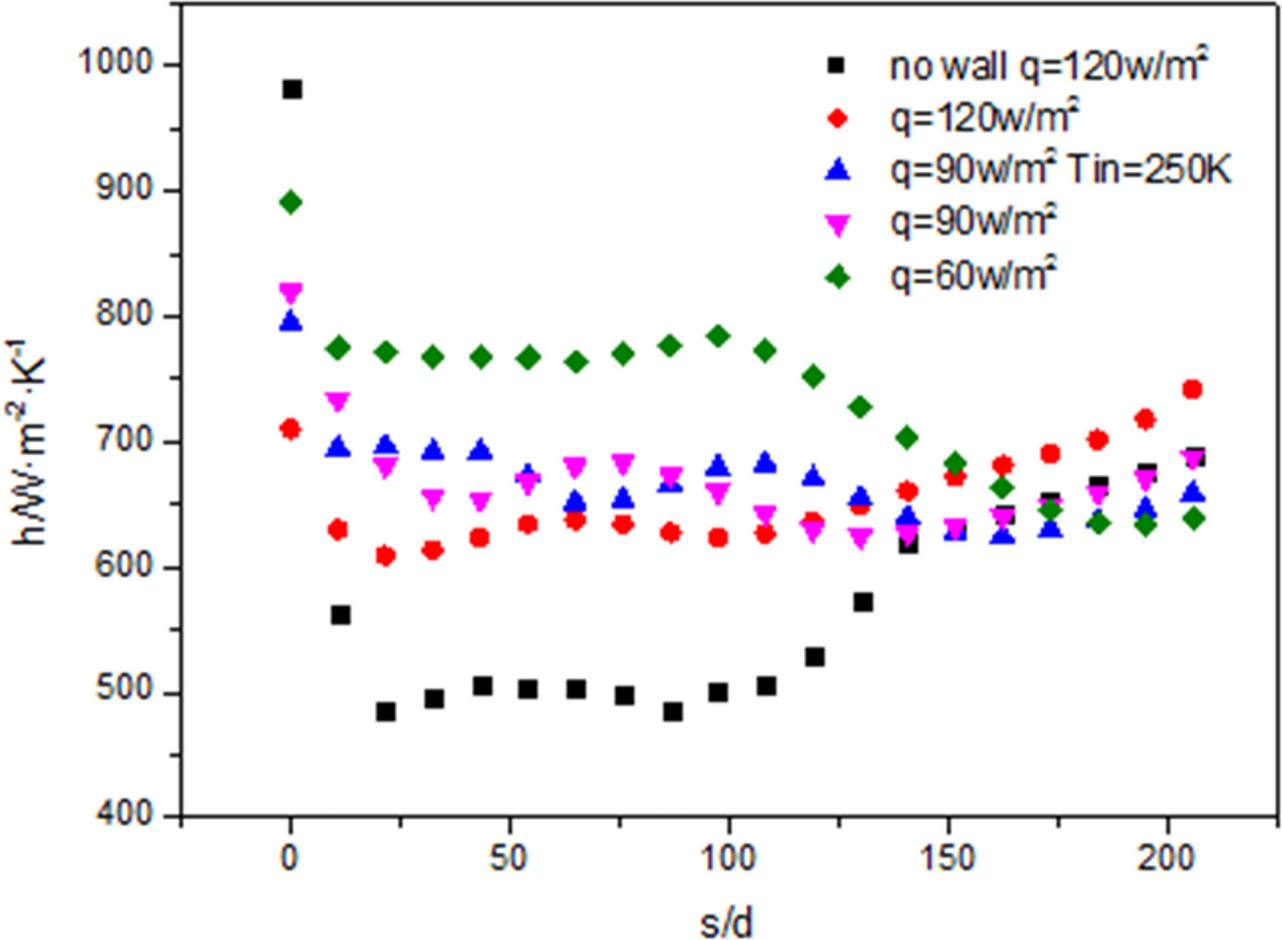
Comparisons of heat transfer coefficient along the tube.

Fig 18 shows the heat transfer coefficient at different heat flux at the same length (L = 1300mm), diameter (d = 6mm), inlet temperature (283K) and mass flow rate (200kg/m^2^s). In the beginning period, the larger heat flux, the smaller the heat transfer coefficient. This is consistent with [2]. Then heat transfer coefficient at higher heat flux increases gradually along the flow direction of the tube, after s/d = 150, it happens that the higher heat flux, the larger the heat transfer coefficient. At the same time the inlet temperature of fluid does not have a significant impact on the heat transfer coefficient. At the beginning the heat transfer coefficient without wall is lower than that with the wall seriously. With the increase of the length, the heat transfer coefficient without wall increases gradually, but still less than that with the wall.

**Fig 19:**
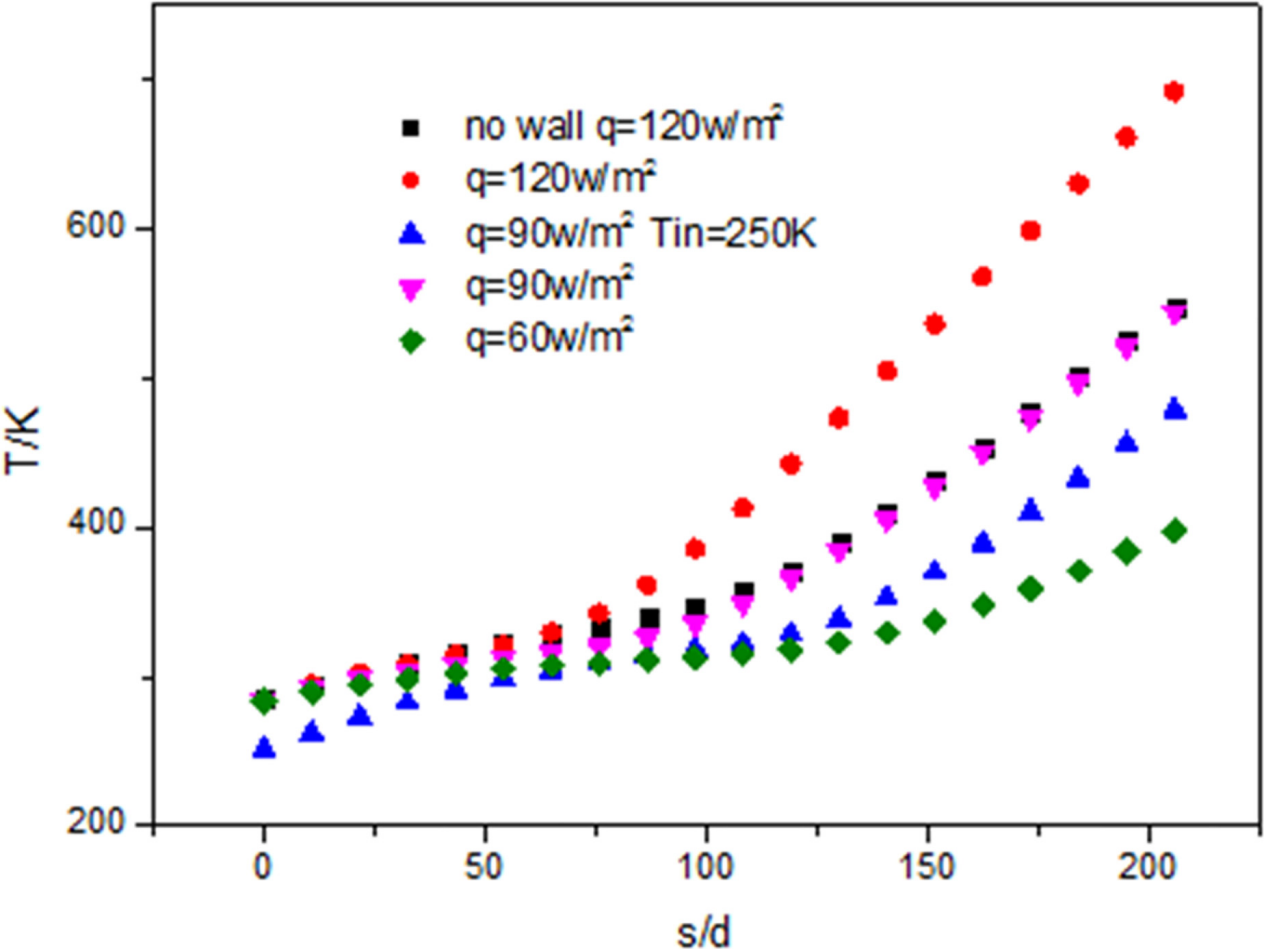
Development of flow temperature (Tf) along the tube.

Fig 19 shows the fluid temperature increases along the flow direction, the higher the heat flux is, the wider the heating temperature range is. Though the inlet temperature is different, the fluid temperature curves at different inlet temperature are almost parallel and the temperature difference between the inlet and out temperature is almost the same. The fluid temperature without wall is lower than that with the wall.

**Fig 20:**
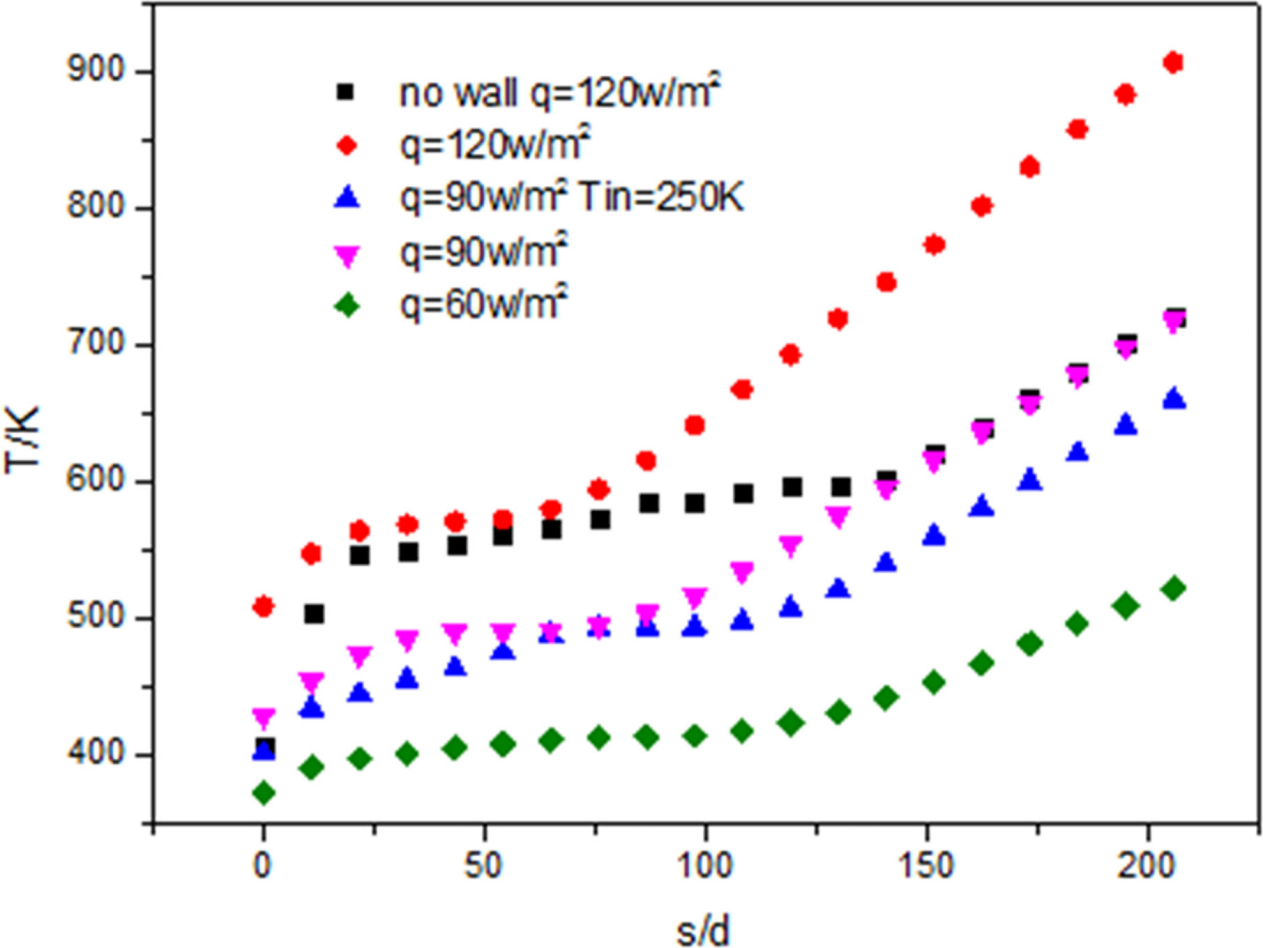
Development of wall temperature (Tw) along the tube.

Fig 20 shows that along the flow direction, with the increase of heat flux, wall temperature gradually increases and the higher the heat flux is, the bigger the wall temperature is. The wall temperature curves at different heat flux are almost parallel. The wall temperature without wall is lower than that with wall.

**Fig 21:**
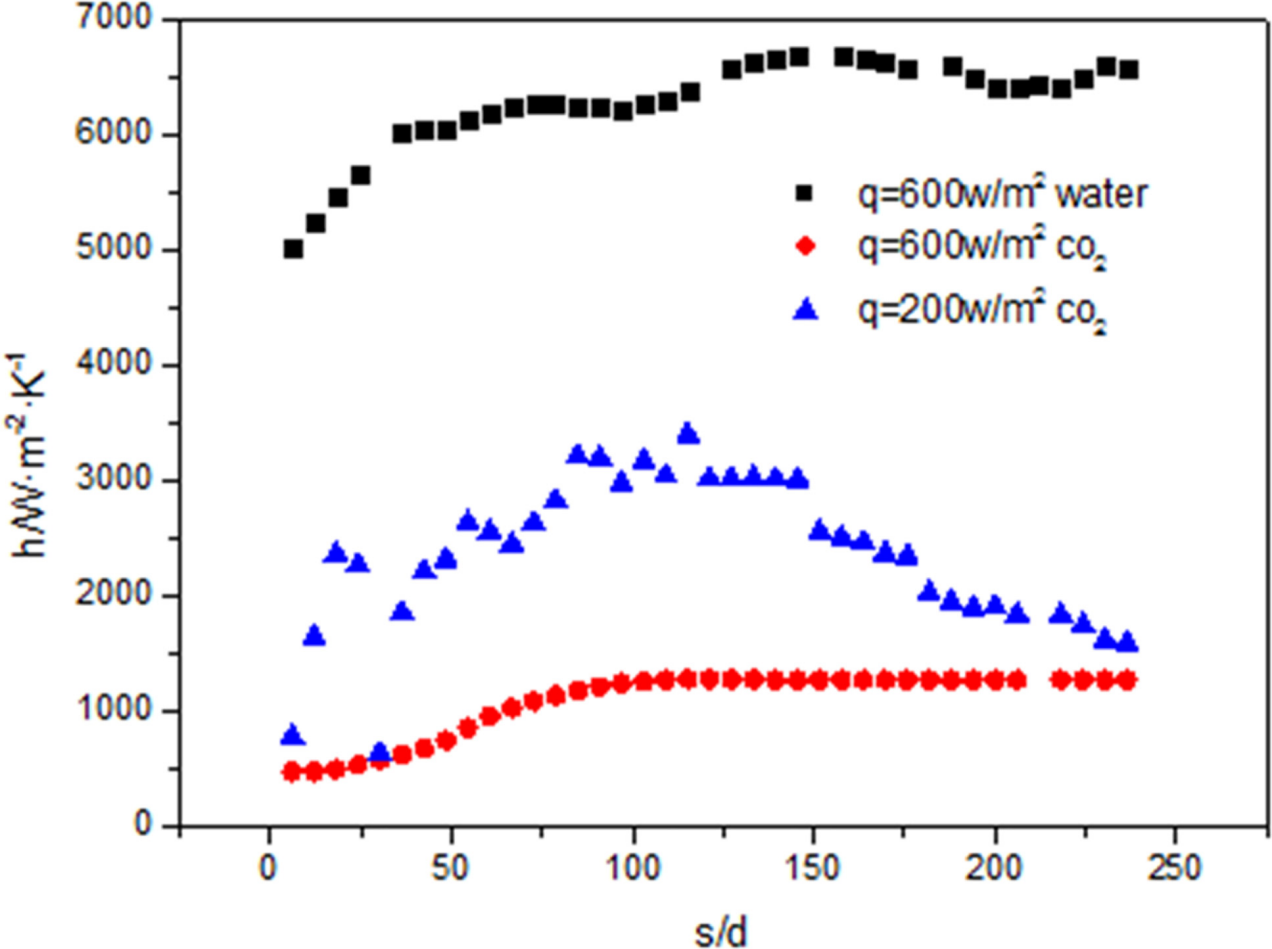
Comparisons of heat transfer coefficient along the tube.

Fig 21 shows when the diameter is 26mm and the mass flow rate is 600kg/m^2^s, the deterioration of heat transfer will happen with increase of the heat flux. When the heat flux is 200kw/m^2^, the change range of the heat transfer coefficient is from 2000 W/m^2^ k to 3000 W/m^2^ k. When the heat flux is 600kw/m^2^, the heat transfer coefficient changes around 1000 W/m^2^ k and it is significantly less than the value at 200kw/m^2^.

Fig 21 shows the heat transfer coefficient of the supercritical water at 25MPa is around 6000 W/m^2^ k and the heat transfer coefficient of CO_2_ under the same flow rate and heat flux is around 1000 W/m^2^ k, which is significantly lower than that of the heat transfer coefficient of water. Because the specific heat capacity of water is significantly larger than that of CO_2_.

The figure (S1 Fig) shows the fluid and wall temperature gradually increase at 200kw/m^2^ along the flow direction of tube. When the heat flux is 600kw/m^2^, the wall temperature along the tube greatly decreases from 1800K to 1000K and then gradually increases to 1800K. The fluid temperature along the tube greatly increases from 250K to 1200K, and the change ranges are large.

The figure (S2 Fig) and the figure (S3 Fig) show the changes of heat transfer coefficient are not obvious along the flow direction of the tube, and the wall surface temperature and the fluid temperature curve are almost coincident when the inlet temperature is different.

## 4. Conclusion

The heat transfer of supercritical CO_2_ in the horizontal straight tube is studied by using numerical calculation method. The following conclusions are obtained:

1. Almost all models are able to present the trend of heat transfer qualitatively, and the stand *k*−*ε* with enhanced wall treatment model shows the best agreement with the experimental data, followed by LB low Re turbulence model.
2. The maximal velocity, maximal fluid temperature and the minimal turbulence kinetic energy locate at the top zone, which demonstrates that flow temperature of the top zone is higher than that of the bottom zone.
3. After the critical temperature, the heat transfer coefficient with wall is greater than that without the wall. The change of heat flux does not affect maximum value of the heat transfer when the diameter and the mass flow rate are kept constant and the heat flux will only change the cooling temperature range. When the mass flow rate is proportion to the heat flux, the heat transfer coefficient achieves the maximum values at the same position along the flow direction. The wall temperature under higher heat flux is lower than that under lower heat flux at the same position along the flow direction. It is the same as that when the length is proportion to the heat flux. When the heat flux is proportional to the inlet fluid temperature, the maximum of heat transfer coefficient is the same at different heat flux. When the heat flux is proportional to the diameter of tube, the heat transfer coefficient which is higher should be calculated.
4. The heating heat transfer of CO_2_ is different from the cooling case. When the heat flux increases to 600kw/m^2^, the deterioration of heat transfer will occurs. In the same condition, the heat transfer of supercritical CO_2_ is smaller than that of supercritical water.

## Supporting Information

S1 Fig Development of wall temperature (Tw) and flow temperature (Tf) along the tube (DOCX)

S2 Fig Comparisons of heat transfer coefficient at different inlet temperature along the tube (DOCX)

S3 Fig Development of wall temperature (Tw) and flow temperature (Tf) at different inlet temperature along the tube (DOCX)
