# Supplementary material for: Numerical Study on the Heat Transfer of Carbon Dioxide in Horizontal Straight Tubes under Supercritical Pressure
Source: PLoS One. 2016 Jul 26;11(7):e0159602. doi: 10.1371/journal.pone.0159602 (PMC4961450; doi:10.1371/journal.pone.0159602)
Supplement: S3 Fig — (DOCX) [file pone.0159602.s003.docx]

S 3 Fig. Development of wall temperature (Tw) and flow temperature (Tf) at different inlet temperature along the tube
